# Chaotropic Ions Mediated Polymer Gelation for Thermal Management

**DOI:** 10.1002/advs.202405077

**Published:** 2024-07-03

**Authors:** Chenxiao Yin, Jingrui Sun, Chang Cui, Ke‐Ke Yang, Ling‐Ying Shi, Yiwen Li

**Affiliations:** ^1^ College of Polymer Science and Engineering State Key Laboratory of Polymer Materials Engineering Sichuan University Chengdu 610065 China; ^2^ The Collaborative Innovation Center for Eco‐Friendly and Fire‐Safety Polymeric Materials (MoE) National Engineering Laboratory of Eco‐Friendly Polymeric Materials (Sichuan) State Key Laboratory of Polymer Materials Engineering College of Chemistry Sichuan University Chengdu 610064 China

**Keywords:** gelation, phase change materials (PCMs), phase change salogels (PCSGs), salt hydrates, thermal management

## Abstract

Energy and environmental issues have increasingly garnered significant attention for sustainable development. Flexible and shape‐stable phase change materials display great potential in regulation of environmental temperature for energy saving and human comfort. Here, inspired by the water absorption behavior of salt‐tolerant animals and plants in salinity environment and the Hofmeister theory, highly stable phase change salogels (PCSGs) are fabricated through in situ polymerization of hydrophilic monomers in molten salt hydrates, which can serve multiple functions including thermal management patches, smart windows, and ice blocking coatings. The gelation principles of the polymer in high ion concentration solution are explored through the density functional theory simulation and verified the feasibility of four types of salt hydrates. The high concentration chaotropic ions strongly interacted with polymer chains and promoted the gelation at low polymer concentrations which derive highly‐stable and ultra‐moisturizing PCSGs with high latent heat (> 200 J g^−1^). The synergistic adhesion and transparency switching abilities accompanied with phase transition enable their smart thermal management. The study resolves the melting leakage and thermal cycling stability of salt hydrates, and open an avenue to fabricate flexible PCM of low cost, high latent heat, and long‐term durability for energy‐saving, ice‐blocking, and thermal management.

## Graphical abstract

The gelation principles of in situ polymerized hydrophilic polymers in molten salt hydrates are demonstrated to fabricate flexible and shape‐stable phase change salogels with low cost, high latent heat, and long‐term durability, inspired by Hofmeister theory and water absorption behavior of euryhalinity species, which present great application potentials for energy‐saving, ice‐blocking, and thermal management.

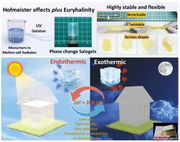

## 1 Introduction

Energy and environmental issues have become worldwide focus because they will finally threaten the survival of humans. Phase‐change materials (PCMs) have been used to regulate temperature for achieving thermal comfort of human life and to improve the thermal energy utilization efficiency thereby relieving the energy crisis.^[^ ^1^, ^2^, ^3^, ^4^ ^]^ Salt hydrates consisted of inorganic salts and water are one promising class of solid–liquid latent heat storage PCMs with the signatures of the easy availability, low cost, desired melting temperature range, and relatively high latent heat.^[^ ^5^, ^6^ ^]^ However, the salt hydrates PCMs may suffer from incongruent melting, weathering, and melting leakage in commercial utilization.

Molten salt hydrates (MSHs) are media whereby the water‐to‐salt molar ratio is close to the coordination number of the strongest hydrating cation and thus contain high concentrations of inorganic ions dissolved in water. MSHs have increasingly been used as deep eutectic “green” solvents^[^ ^7^, ^8^, ^9^ ^]^ with features similar to traditional ionic liquids (ILs), e.g. high ionic environments, low melting point, ionic conductivity, non‐volatility, and thermal stability.^[^ ^10^, ^11^ ^]^ The emerging method of polymer gelation in MSHs aims to achieve form‐stabilization,^[^ ^12^, ^13^, ^14^, ^15^ ^]^ for example, Sukhishvili et al. reported the poly(vinyl alcohol) dissolved and gelated in LiNO_3_⋅3H_2_O (LNH) for generate salogels with sol–gel transition behavior to control the fluidity.^[^ ^12^, ^13^, ^14^ ^]^ However, the dissolving and gelation of polymer in high‐salt aqueous environments of MSHs still face great challenges, and the gel–sol transition still leads to melting leakage. It has been demonstrated that the ability of salts to precipitate proteins and hydrophilic polymers from an aqueous solution follows the Hofmeister series,^[^ ^16^, ^17^ ^]^ which can be divided into “kosmotropic” and “chaotropic” effects.^[^ ^18^ ^]^ Therein, “chaotropes” disrupt water structure and directly interact with polymer chains through the salinization pathway, increasing the surface tension of the water/hydrophobic interface and leading to chain swelling.^[^ ^16^, ^17^ ^]^ However, this foundational understanding of the interactions between salt ions and polymers were conducted in aqueous salt solutions with salt concentration much lower than that of MSHs (Table S1, Supporting Information).^[^ ^19^, ^20^, ^21^, ^22^ ^]^

**Figure 1:**
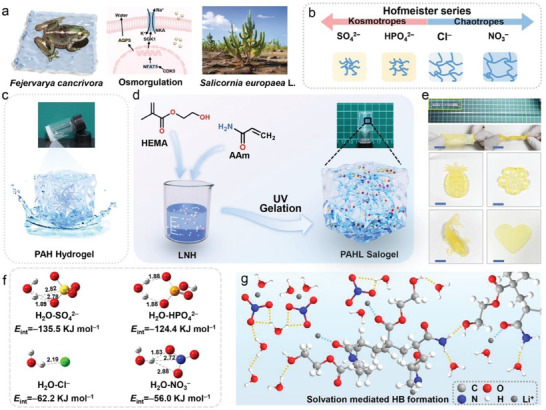
Schematic illustrating the design concept of stable PCSGs. (a) The osmoregulation mechanism of *Fejervarya cancrivora* (left) and *Salicornia europaea* L. (right) through transport/channel proteins in high saline environments (middle). (b) Hofmeister series with different cations and anions and the models of polymer chains in contraction and expansion in corresponding salogels. (c) The paragraph and diagram of PAH hydrogel. (d) The paragraph and diagram of PAHL salogel. (e) PAHL salogels can be stretchable and twistable, and easy to make into various shapes, e.g. pineapples, flowers, koi carp, and heart (Hydrophilic fluorescent yellow dye was added to the samples for better imaging). (f) DFT molecular simulation results of the interaction energy (*E*_int_) for H_2_O‐SO_4_^2−^, H_2_O‐HPO_4_^2−^, H_2_O‐Cl^−^, and H_2_O‐NO_3_^−^. (g) The HBs networks of PAHL salogels, where the NO_3_^−^, Li^+^ form hydrogen bonding/coordination interactions with water and polymer chains, promoting the dissolution and gelation of polymer chains in high concentration saline solutions.

In nature, there are few salt‐tolerant animals and plants, e.g. *Fejervarya cancrivora* ^[^ ^23^, ^24^ ^]^ and *Salicornia europaea* L.,^[^ ^25^, ^26^ ^]^ which perform osmotic regulation through a series of transport/channel proteins in high salinity environment (**Figure** **1**a). The strongest salt‐absorbing plant, *Salicornia europaea* L., gather high‐concentration salt in the body and thus enable the root system to absorb water from soil. Inspired by the euryhaline animals/plants and the Hofmeister effect, we proposed to use high salt concentration “chaotropic” MSHs to promote the gelation process of polymerized polymers, not only resolving melting leakage problem but also promoting continuous moisturizing and maintainlong‐term stability.

In this work, we provide the principles of polymer gelation in MSHs to fabricate form‐stable and robust phase change salogels (PCSGs, the gel formed from polymer/salt hydrates was defined as salogel here) through in situ photo‐polymerization of hydrophilic monomers dissolved in MSHs overcoming the gravitational settling of polymer solids in the low viscous MSHs. The density functional theory (DFT) simulation, the Raman and infrared spectroscopy were conducted to reveal the interaction among the ions, water, and the polymer chains. The coordination and electrostatic interaction within the network not only improve the gelation process at an ultralow polymer content, but also solves the melting leakage and improve the thermal stability of salt hydrates while retaining the high enthalpy. Meanwhile, the high‐concentration saline environment promotes the water‐absorption and moisturizing of salogel system prohibiting water loss in the long‐term application. In addition, we verify this scenario over several kinds of salt hydrates and monomers for fabricating the PCSGs. We exploited the thermal, form‐stable, mechanical, shear adhesion, frost‐resistant performances of the PCSGs, and demonstrated their thermal management and ice‐blocking application. We believe this study could offer new opportunities toward the fabrication of robust flexible salogels of low cost, high latent heat and long‐term durability for energy‐saving and thermal management.

## 2 Results

### 2.1 Fabrication of Phase Change Salogels and Interaction‐Solvation Analysis

According to the Hofmeister series (Figure 1b), we choose the chaotropic LiNO_3_⋅3H_2_O (LNH) and CaCl_2_⋅6H_2_O (CCH) MSHs as the solvents, and the kosmotropic Na_2_SO_4_⋅10H_2_O (SSD) and Na_2_HPO_4_⋅12H_2_O (DPDH) for comparison. We found that the polymerized P(AM‐*co*‐HEMA) (PAH) hydrogel (Figure 1c) from 0.2 g monomers with weight ratio of AM and HEMA ≈1:1 cannot absorb 2 g water and almost half of the water were drain out of the gel. When the water is replaced by the chaotropic MSHs LNH, e.g. the polymerization of 0.2 g monomers dissolved in 2 g molten LNH PCMs produced form‐stable PAH/LNH (PAHL) salogels (Figure 1d,e).

In order to explore the mechanism of gelation, the interaction energy (*E*_int_) among the anions, water and polymer chains were calculated through DFT simulation, and the binding energy *E*_int_ between the anions and water was in the order of SO_4_^2−^ > HPO_4_^2−^ > Cl^−^ > NO_3_^−^ (Figure 1f) aligned with the Hofmeister series of anions.^[^ ^16^, ^17^ ^]^ The Hofmeister effects of cations were in order of Na^+^> Li^+^ > Ca^2+^, thus, both anions and cations of SSD and DPDH preferentially bind water and weaken the hydrogen bonding between water and −CONH_2_, −OH, leading to dehydration of the polymer chains (Figure S1a, Supporting Information). In contrast, chaotropic ions LNH and CCH increase the surface tension of the water/hydrophobic interface and the ions tightly bind with −CONH_2_ and −OH, increasing the solvation of PAH chains in MSHs (e.g. Figure 1g illustrates the solvation of PAH in LNH) and generated stable salogels at low polymer concentrations (Figure S1a, Supporting Information). We also demonstrated the gelation behavior of P(AM‐*co*‐AA) and P(AM‐*co*‐AN) in the LNH (Figure S1b,c, Supporting Information). The copolymerization of different monomers can tune the loading ratio of MSH and the mechanical performance of the salogels, and in particular for the AM‐HEMA system, the ion coordination interaction of Li^+^ with C═O and OH on HEMA units and with ─C(═O)NH_2_ on AM units, the hydrogen bonding interaction of ions and polymer chains as well as the chain entanglement ensure the high stability of the salogels. Given high enthalpy of the LNH and the facile polymerization to generate high mechanical performance P(AM‐*co*‐HEMA)/LNH (PAHL) salogels, we mainly studied a series of stable PAHL salogels by adjusting the composition of monomer and salt hydrates in the solution (Tables S2 and S3, Supporting Information) in the following text, where PAHL*_y_* (*y* = 1−10, Table S3, Supporting Information) indicated the salogels with different monomer contents in Table S2 (Supporting Information), and PAHL_4_W*_x_* (*x* = 1−4) indicate the salogels with additional water in Table S3 (Supporting Information). Small amount of additional water aims to reduce the water binding competition of the polymer from salt hydrates and thus tune the phase change temperature and enthalpy. The prepared molten salogels can be stretched and twisted, making it is easy to produce samples with various complex shapes (Figure 1e) in large volumes (Figure S2, Supporting Information) to meet customization needs.

**Figure 2:**
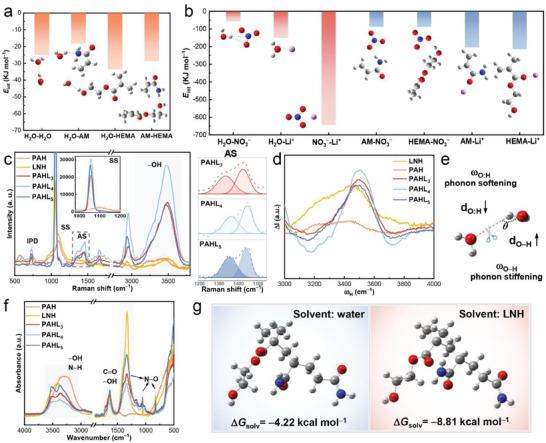
Theoretical and experimental analysis of the interactions within PAH hydrogels and PAHL salogels. The molecular simulation for the interaction energy (*E*_int_) (a) among water and monomer units of PAH chain and (b) among the molecules and ions in PAHL salogels. (c) Raman spectra and (d) Raman Differential Phonon Spectrometrics. (e) Schematic showing HB breakage between two water molecules, leading to an elongation of the O:H non‐bond and shortening of the O−H polar bond, representing a softening of the O:H phonon and stiffening of the O−H phonon. (f) FTIR spectrum. (g) The solvation free energy (Δ*G*_solv_) of PAH polymer chains in water and in LNH from DFT simulation, respectively.

To explore the detailed interaction among the components in the PAHL salogel, we calculated the binding energy (*E*_int_) among the water and monomer units (**Figure** **2**a) as well as that between these molecules and ions (Figure 2b). The DFT calculation proved that the *E*_int_ of Li^+^‐H_2_O and NO_3_^−^‐H_2_O^[^ ^27^, ^28^ ^]^ was higher than the *E*_int_ of H_2_O–H_2_O in the LNH system. Therefore, in molten LNH, the water molecules interact with Li^+^ and NO_3_^−^ in the form of solvation shells (Figure S3, Supporting Information). Moreover, the Li^+^ and NO_3_^−^ own a stronger interaction (higher *E*_int_) with AM and HEMA units of the polymer chains (Figure 2b) than that between the PAH polymer chains themselves (Figure 2a) enabling the Li^+^ and NO_3_^−^ ions to insert into the polymer chains for breaking the HBs between the polymer chains and regulating the mechanical flexibility of the salogels.

The solvation of PAH polymer chains and their interaction with abundant inorganic ions in LNH were confirmed by Raman spectra, where the nitrate NO_3_^−^ is Raman active. The perturbations from metal cations and water molecules as well as the polymer chains result in changes in the symmetry of NO_3_^−^, contributed to more vibrational bands (Figure 2c; Figure S4, Supporting Information).^[^ ^29^ ^]^ Compared to LNH and PAH, the PAHL salogels presented increased intensity and decreased bandwidth of the symmetric stretching (SS) band at 1049 cm^−1^ (Table S4, Supporting Information) indicating a decrease in the form of contact Li^+^‐NO_3_^−^ ion pairs (because ion‐paired nitrate species relax faster than the free species), reflecting the NO_3_^−^ interact with polymer chains in addition to H_2_O. The splitting of the asymmetric stretching (AS) band at 1370 cm^−1^ confirms the disturbance of water molecules and the long‐range Coulomb disturbance of Li^+^.^[^ ^29^ ^]^ The AS band can be deconvoluted into two peaks (Figure 2c, right), where changes in −OH and −CONH_2_ content affect the hydration of NO_3_^−^ and the Coulomb effect between Li^+^‐NO_3_^−^. The Raman differential phonon spectra (DPS)^[^ ^30^, ^31^ ^]^ (Figure 2d) generated from normalizing the Raman spectra (Equation (1))^[^ ^31^, ^32^ ^]^ further demonstrate that the influence of the salt ions on Coulombic interactions and polarization is similar to heating, softening O:H phonons and hardening O−H phonons, effectively disrupting the HBs network of water (Figure 2e) and PAH chains. Fourier transform infrared (FTIR) spectra (Figure 2f; Figure S5, Supporting Information) further suggest the change in dipole moment related to the N−O stretching vibration of NO_3_^−^ in PAHL at 825, 1056, and 1328 cm^−1^,^[^ ^33^ ^]^ and the intermolecular interaction between solutes and polymer chains strongly affects the tensile vibration of −OH (3000−3360 cm^−1^) evidenced by the significant splitting of the −OH tensile vibration peak in PAHL. We also observed the tensile vibration of N−H in AM units (3400−3530 cm^−1^) of PAH and PAHL.

We calculated the solvation‐free energy (Δ*G*_solv_) (Figure 2g) of PAH in LNH which is even lower than that in pure water, indicating Li^+^ and NO_3_^−^ promote the strong solvation of PAH chains in water. Moreover, Li^+^ has been demonstrated to be coordinated with the O and N atoms on the −C(═O)O−, −OH and −C(═O)NH_2_ groups, and the NO_3_^−^ has been demonstrated to form multiple HBs with the −CONH_2_, both of which play as the physical crosslinking agents promoting the gelation process.

### 2.2 Thermal Properties and Thermal Stability of PAHL

**Figure 3:**
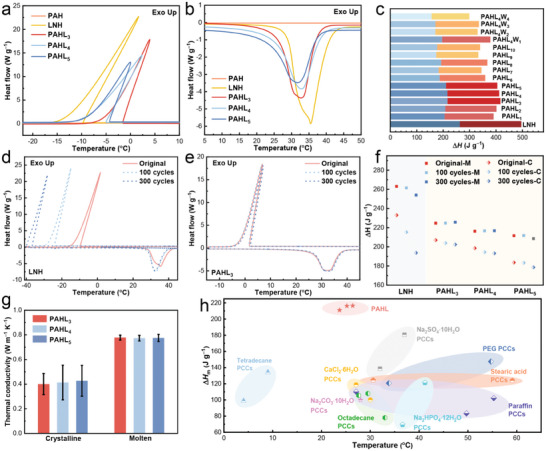
The characteristics of thermal performance and thermal stability for LNH, PAH, and PAHL. The DSC curves during (a) cooling and (b) heating process. (c) Phase change enthalpy of LNH, PAHL, and PAHLW. (d) DSC curves showing severe undercooling and inconsistent melting of LNH during thermal cycling. (e) The DSC curves of PAHL_3_ almost overlap after 300 thermal cycles. (f) The comparison of phase change enthalpy after thermal cycles. (g) Thermal conductivity of PAHL in different state. (h) The comparison of latent heat of low‐temperature form‐stable PCCs (*T*_m_ = 5−60 °C) between PAHL_3−5_ (this work) and recent literature.^[^ ^35^, ^36^, ^39^ ^]^

The thermal properties of PAHL and PAHLW salogels were characterized by differential scanning calorimetry (DSC) (**Figure** **3**a,b; Figure S6, Supporting Information) and the critical parameters are summarized in Figure 3c and Tables S5 and S6 (Supporting Information). The melting temperature (*T*_m_) and crystallization temperature (*T*_c_) of the just prepared LNH (supporting information) were ≈28.9 °C and −8.8 °C, respectively, and the melting latent heat (Δ*H*_m_) was ≈263.1 J g^−1^ which were slightly lower than the reported values (*T*_m_ ≈29.2–30.1 °C, Δ*H*_m_ = 273–296 J g^−1^)^[^ ^34^ ^]^ presumably due to the deviation in the water concentration. Compared with the LNH, the *T*_m_ of PAHL decreased and the *T*_c_ increased, e.g. PAHL_3_ with *T*_m_ at 26.4^ o^C and *T*_c_ at −3.1 °C, PAHL_4_ with *T*_m_ at 25.2 °C and *T*_c_ at −4.2 °C, and PAHL_5_ with *T*_m_ = 23.7 °C and *T*_c_ = −3.8 °C, indicating that the interactions between PAH polymer chains and Li^+^ and NO_3_^−^ alleviate the supercooling. It is worth noting that the DSC measurements with small sample volumes presented increased supercooling due to the volume scaling dependence of crystal nucleation and the real crystallization of the PAHL salogels occurred at ≈10 °C (Figure 5d). High phase change latent density (Δ*H*) is the premise to the thermal management of the PCMs. The encapsulation methods to fabricate the form‐stable phase change composites (PCCs) always sacrifice part of the enthalpy and lead to the decrease of Δ*H*.^[^ ^35^, ^36^ ^]^ The Δ*H* of PAHL (e.g. PAHL_3_, Δ*H*_m_ = 216.5 J g^−1^) here can reach 84% of the initial prepared LNH (Δ*H*_m_ = 263.1 J g^−1^), while the Δ*H* of PAHLW reaches 60−76% of LNH due to that the additional water tends to dissolve/solvate the salt.^[^ ^37^ ^]^ The enthalpy density of most salogels still reaches up to 200 J g^−1^.

Interestingly, the gelation of the salogels in MSHs greatly increased thermal stability and prevented the increase of supercooling during long‐term thermal cycling. For the pure LNH, after thermal cycling, the supercooling increased and the latent heat slightly decreased (Figure 3d,f). Nevertheless, the DSC curves of PAHL after multiple thermal cycles almost coincide with the original one (Figure 3e; Figure S7, Supporting Information), and the Δ*H* only changes slightly (Figure 3f), demonstrating that the hydrogen bonding and coordination interaction between −CONH_2_/−OH of polymer chains and LNH contributed to the homogeneous salogels with improvement of the thermal cycling stability without increase of supercooling of LNH.

Generally, salt hydrates possess higher specific heat capacity and higher thermal conductivity than organic PCMs, and thus promise for heat storage applications. The thermal conductivity of PAHL during melting and crystallization is as high as 0.78 and 0.43 W m^−1^K^−1^, respectively (Figure 3g), which are comparable and even better than some organic PCCs with thermal conductive fillers (such as the n‐alkane impregnated in Cellulose/CNTs aerogels ≈0.58 W m^−1^K^−1^ and PEG impregnated in Diatomite/CNTs ≈0.29 W m^−1^K^−1^).^[^ ^35^, ^38^ ^]^ Compared with the low‐temperature PCCs reported in recent literature,^[^ ^35^, ^36^, ^39^ ^]^ the thermal performance of PAHL in this work has significant advantages for temperature management (Figure 3h).

In addition to the thermal stability, our salogels can retain the water under both high and low temperature in dry environment, overcoming the water loss shortage of the general hydrogels.^[^ ^40^, ^41^ ^]^ The interactions between water molecules and salt ions reduced the volatilization of water,^[^ ^42^ ^]^ and the high ion environment as well as the crystalline‐melting process enable the water absorption from environment.^[^ ^43^ ^]^ We recorded the liquid retention of the salogels by periodically weighing the gels stored under ambient and dry conditions for 100 days (Figure S8a–d, Supporting Information). We found that PAHL is hygroscopic at ambient conditions and can absorb a certain amount of water and the water absorption reaches an equilibrium in several days (Figure S8c, Supporting Information) and the resulted salogels should behave like the PAHLW, and the water absorption of PAHL_4_W_4_ with the additional water accounting 20 wt.% of LNH was <5% (Figure S8e, Supporting Information) and the latent heat of the PAHL_4_W_4_ was still higher than 150 J g^−1^ (Table S6, Supporting Information). In case the application of PAHL in high humidity environment, the flexible water‐resisting polymer films can be used as encapsulation layer to main the high enthalpy. In the dry environment with RH 14%, the weight loss of the PAHL was <2 wt.% in 100 days (Figure S8d, Supporting Information), and the weight loss increased with the increase of additional water content in the PAHLW (Figure S8f, Supporting Information). The improvement of evaporation resistance is attributed to the low equilibrium vapor pressure of LNH and the strong interaction of the water with ions and polymer chains in the gel network. The evaporation resistance also ensures long‐term stability during application.

### 2.3 Mechanical Property and Shear Adhesion Performance

**Figure 4:**
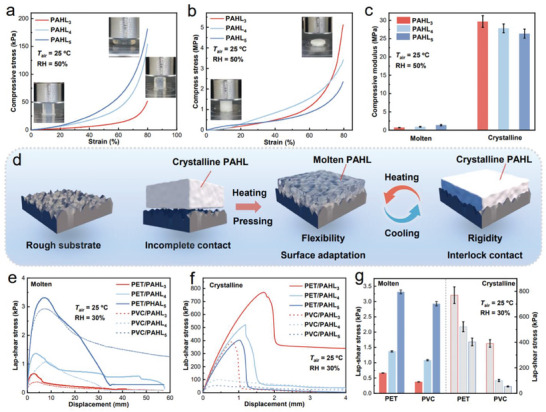
The mechanical properties and shear adhesion performance of PAHL salogels. The compression stress–strain curves of PAHL_3−5_ samples in (a) molten and (b) crystalline state. (c) The elastic modulus compression of PAHL_3−5_ samples in molten and crystalline state. (d) Lap‐shear stress versus displacement curves of PAHL_3−5_ samples in (e) molten and (f) crystalline state on different substrates. (g) The adhesion strength comparison of PAHL_3−5_ samples in molten and crystalline state.

Salt ions have been employed in tailoring mechanical and anti‐freezing properties of hydrogels.^[^ ^44^, ^45^ ^]^ In our work, the PAHL salogels with ultralow polymer weight ratio have shown suitable mechanical strength, for instance, the PAHL_3_ can be reversibly elongated more than 6 times of its original length (Figure 1e). The rheological frequency scan (Figure S9, Supporting Information) curves always show *G*’ > *G*’’ of PAHL samples before and after LNH melting, and salogels heated on thermostatic platform also present excellent form‐stability (Figure S10, Supporting Information), indicating that the PAH polymer network can effectively prevent the leakage of molten LNH. **Figure** **4**a,b, shows the compression curves of the PAHL curves in molten and crystalline state in which the highest compression strain was set at 80%. The compressive elastic modulus of the molten salogels increased in the range of 0.15−1.8 MPa with the increase of the HEMA mole ratio in monomers (Figure 4a,c; Figure S11, Supporting Information). The compressive modulus of crystalline PAHL_3_ was high up to 29.7 MPa, increased by ≈30 times of molten salogels (Figure 4b,c). When the compressed crystalline sample was heated to be above 26 °C, it recovered to the original shape, and the elasticity of PAHL was restored (Figure S12, Supporting Information).

The PAHL salogels show adhesive characteristics in response to temperature, i.e., *T* > *T*_m_, the salogels are pressure sensitive and show surface adaptability (Figure 4d), and the adhesion force between the PAHL and substrate solidify after crystallization. The lap‐shear adhesion test on the PAHL samples sandwiched between two substrates obtained the stress displacement curves and adhesion strength of PAHL samples in the molten and crystalline states (Figure 4e–g), respectively. The shear bonding strength of the PAHL_5_ in molten state is 0.5−3.3 KPa on the hydrophobic substrate (e.g. PET and PVC sheets, with similar surface energy of PMMA which are used for making sunrooms for thermal management later) and the flexible PAHL samples show an elongation before spalling (Figure S13a, Supporting Information). After crystallization, the adhesion strength sharply increased which reach 769.6 KPa for the PAHL_3_ on PET, without deformation during spalling (Figure S13b, Supporting Information). The crystallization‐induced curing process significantly improves the shear adhesion strength between PAHL and substrate, whereas the flexible PAHL adhesive is easy to form stress concentration and then evolve into the bonding failure point (Figure S13a, Supporting Information). Overall, the higher polymer content induced higher compressive modulus and stronger adhesion strength for molten salogels, and the higher salt hydrates content contributed to higher compressive modulus and stronger adhesion strength for crystalline salogels.

### 2.4 Thermal Management and Ice Blocking Applications

**Figure 5:**
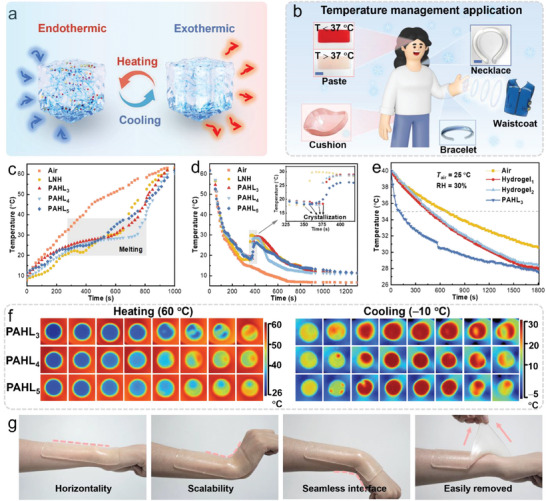
The temperature management of the PAHL salogels for human comfort. (a) Schematic illustration of exothermic and endothermic phenomenon during the phase change process of salogels. (b) Temperature management products, e.g. paste (added hydrophilic variable temperature particles, scale bar: 2 cm), necklace (scale bar: 5 cm), cushion, bracelet, and waistcoat. The time‐temperature curves during (c) heating and (d) cooling process. (e) Apply PAHL paste to the outer wall of a beaker containing 100 mL of 40 °C water and compare the cooling process with other hydrogel pastes. (f) The infrared thermal images of temperature varieties for PAHL samples on the 60 °C thermostatic platform and −10 °C cooling platform. (g) A piece of stretchable PAHL paste can fit on a human wrist even under a large tilting action.

The form‐stability of the PCMs allows the reversible endothermic and exothermic process during the crystallization and melting (**Figure** **5**a). The suitable phase change temperature (*T*_m_ ≈26 °C and *T*_c_ ≈10 °C) and high heat capacity of PAHL allow the potential human comfort temperature management, e.g. antipyretic pastes, cooling necklace, ice cushion, waistcoat, etc. (Figure 5b). We evaluated the smart temperature management capability of PAHL by recording the time‐temperature curves during heating/cooling process (Figure 5c,d) using thermocouple thermometer collected with 10 mL bottle containing air, 5 g of LNH, or 5 g PAHL put in a water bath, respectively. The temperature variations of air, LNH, and PAHL_3‐5_ were recorded and compared during water bath heating and ice–water bath cooling processes. During the heating process of LNH and PAHL (Figure 5c), a temperature rise region with a slow rising rate occurred corresponding to the melting of LNH. Even when the air was heated to 45 °C, the PAHL was still maintained at ≈28 °C. During the cooling process, a crystallization exothermic induced a temperature recalescence, ≈15 °C higher than the air temperature (Figure 5d). When the PAHL paste with the size 120 mm × 50 mm × 2 mm in length × width × thickness was sticked around the outer wall of a beaker containing 100 mL of 40 °C, the temperature of the water can quickly decrease to 35 °C (t ≤ 100 s), and the temperature cooling rates was faster than that using two commercially hydrogel antipyretic pastes with the same size (t = 460 and 480 s, respectively) (Figure 5e). Specially, by adding the temperature responsive nanoparticles (*T*_trans_ = 37 °C) to the PAHL paste (Figure 5b), it can realize real‐time temperature sensing for cooling and fever detection, i.e., when the temperature is higher than 37 °C, it will turn white indicating that the patient has a continuous fever and the antipyretic pastes should be changed, and the paste can be reused again after cooling crystallization stored in the refrigerator. The temperature changes of melting endothermic and crystallization exothermic were also observed from the infrared thermogram (Figure 5f; Figure S14, Supporting Information). The PAHL paste shows good scalability under various large bending strains and can achieve seamless interface adhesion with the human wrist, and it can still be easily removed from the finger without causing tissue damage (Figure 5g).

**Figure 6:**
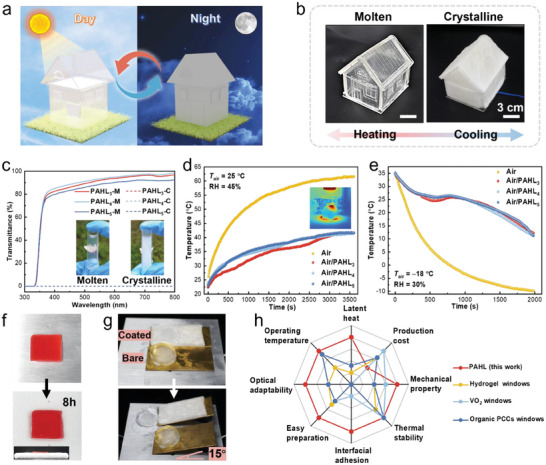
Sunroom thermal management and ice‐blocking coatings application of PAHL salogels. (a) Schematic showing absorbing heat and melting into transparent to alleviate excessive temperature inside the sun room during the day; Cooling and crystallization with heat release to protect privacy at night. (b) The PAHL is transparent in molten state and becomes opaque after crystallization. (c) UV–vis spectra of PAHL in molten and crystalline state. The time‐temperature curves of the sun room covered with PAHL (d) under 100 mw cm^−2^ sunlight and (e) in −20 °C cooling chamber. The blank control group is an uncovered sun room. (f) PAHL was placed on the cold platform (Peltier temperature, *T*_pel_ = −18 °C) for 8 h without any condensation‐frosting, while the frost thickness on the cold platform reached ≈20 mm (*T*_air_ = 25 °C, RH = 38%). (g) Due to the exothermic crystallization of PAHL, the contact area of the ice blocks melts. Tilt the copper plate 15°, and the ice blocks on the copper plate are fixed, while the ice blocks on the PAHL coating slide off. (h) The radar plots of the comprehensive performance of different smart windows.^[^ ^46^, ^48^, ^49^ ^]^

More interestingly, the PAHL is a thermally responsive material with tunable optical properties, and it melts and absorbs heat keeping the indoor temperature low and then becomes transparent, and it crystallizes and becomes opaque at low temperatures at night and thus protects user privacy (**Figure** **6**a,b). The salogels can be another building thermal management candidates in addition to the intelligent windows made of thermochromic materials.^[^ ^46^, ^47^ ^]^ The UV–vis spectra (Figure 6c) demonstrate the high transmittance of the PAHL in the molten state and the opaque state in the crystalline state (Figure 6c). We fabricated a sunroom made of PMMA through 3D printing and the PAHL gel was feasibly sticked on the transparent PMMA sunrooms (Figure 6b). Under the irradiation of a solar simulator (110 mW cm^−2^),^[^ ^15^ ^]^ the temperature in the blank sunlight room without the PAHL cover rose to ≈61.6 °C, while the temperature in the sunroom covered with 2 mm thick PAHL was maintained at ≈41.7 °C after 1 h, effectively alleviating the temperature increase (Figure 6d). We further simulated the night under the low‐temperature environment (*T*_air_ = −18 °C, RH = 30%), and the temperature in the uncovered sunroom dropped to −9.8 °C in 33 min, but the temperature in the sunroom covered with PAHL decreased slowly to 11.3 °C at 33 min (Figure 6e), realizing the comfortable heat preservation and the room become opaque for the privacy protection.

Furthermore, the PAHL coatings present frost‐resistant and icephobic performance. Figure 6f illustrates the severe frost formation on bare Al plate surface (*T*_pel_ = −18 °C). In contrast, the PAHL patch did not show condensation‐frost/freezing even after freezing for 8 h in the same condition, indicating excellent frost‐resistance (Figure 6f). This should be ascribed to that the icing process of water on the surface will release heating which will be absorbed by the salogels inducing the melting of the salogels, and then the melted salogels will recrystallize and release heat leading to the melting of the ice on the surfaces. The synergistic effect of surface melting and freezing point reduction allows ice blocks to easily slide off the surface of PAHL, different from anchoring ice blocks on bare substrate (Figure 6g). Compared with various intelligent energy‐saving windows reported in recent literature, PAHL has excellent comprehensive performance in terms of thermal and mechanic performance as well as the adhesion and cost etc. (Figure 6h),^[^ ^46^, ^48^, ^49^ ^]^ along with the frost resistant and ice‐blocking advantages.

## 3 Conclusion

In summary, inspired by the water‐absorption behavior of salt‐tolerant plants and according to classical Hofmeister theory, we demonstrated the gelation principles of hydrophilic polymers in the high‐salt ion concentration solution of the molten “chaotropic” salt hydrates and produced highly‐stable super‐moisturizing phase change salogels (PCSGs). The suitable *T*_m_ (≈20−26 °C) and high latent heat (≈216.5 J g^−1^) of the PCSGs can rapidly cool the 40 °C heating object to 35 °C (t ≤ 100 s), and is suitable for temperature management of human comfort. Accompanying with melting/crystallization phase change of salt hydrates, the PCSGs switch freely between flexible (deformable) and rigid states, simultaneously achieving adhesion enhancement and optical transparency switching, which ensure the advanced thermal management in energy‐saving sunrooms. In the daytime, the PCSGs are endothermically melted to alleviate the temperature rise without blocking the free passage of light. At night, the crystallization‐induced heat release realizes the heat preservation, meanwhile, gels undergo an opaque transition to protect user privacy. Under extremely cold areas of −18 °C, the PCSGs present frost resistance and ice‐blocking performance. Given the generality of this approach, the richness of potential salt hydrates and monomers building blocks, and the range of thermal and mechanical properties that can be achieved, our study may open an exciting area for further systematic exploration of salogels in thermal management, intelligent windows, and ice‐blocking coatings.

## 4 Experimental Section

### Materials

Lithium Nitrate (LiNO_3_, 99%) was purchased from Adamas‐beta and stored in an airtight desiccator at room temperature before usage. Acrylamide (AM, AR, 99.0%) obtained from Shanghai Macklin Biochemical Co., Ltd. 2‐Hydroxyethyl methacrylate (HEMA, AR, 96%), Acrylic acid (AA, AR, 99%), Acrylonitrile (AN, AR, 99%), and 2‐hydroxy‐2‐methylpropiophenone (photoinitiator 1173, 97%) were purchased from Aladdin Industrial China. Sodium sulfate decahydrate (NaSO_4_·10H_2_O, SSD, AR), disodium phosphate dodecahydrate (Na_2_HPO_4_·12H_2_O, DPDH, AR), and calcium chloride (CaCl_2_, AR) were purchased from Sinopharm Chemical Reagent Co., Ltd. Deionized water was used in all experiments.

### Preparation Methods of Salogels

Acrylamide (AM) and hydroxyethyl methacrylate (HEMA) monomers were added into melted lithium nitrate trihydrate (LNH) or lithium nitrate trihydrate aqueous solution (LNHW) and stirred for 30 min until the monomers were dissolved. Then, the photoinitiator 2‐hydroxy‐2‐methylphenylacetone (1173) was added and stirred in the dark environment for 10 min until dissolved. The detailed formulations of the solution are shown in Tables S2 and S3 (Supporting Information). After 1 h polymerization at room temperature under 365 nm UV (light intensity 36 mw cm^−2^), a series of transparent PAHL*_y_* (*y* = 1−10) and PAHL_4_W*_x_* (*x* = 1−4) gels were obtained. Similarly, PAH/SSD, PAH/DPDH, and PAH/CCH salogels were prepared, respectively by using the molten SSD, DPDH, and CCH as solvents. By changing the second monomer to be acrylic acid (AA) and acrylonitrile (AN), the P(AM‐*co*‐AA) and P(AM‐*co*‐AN) salogels were prepared.

### Interaction Analysis by ATR‐FTIR and Raman Measurement

The ATR‐FTIR spectra were measured by Nicolet 6700 (Thermo Fisher Scientific, USA) at ambient temperature (*T*_air_ = 20 °C). The range of wavelength was set as 4000−400 cm^−1^.

Raman spectra were measured by DXR2xi Raman Imaging Microscope (Thermo Fisher Scientific, USA), using a 532 nm laser as the excitation source. A Raman differential phonon spectrometrics (DPS) was described as follows:

(1) $$I_{\mathrm{hydration}}\left(\omega H\right)=I_{\mathrm{solution}}\;\left(\omega H\right)-\alpha I_{\mathrm{water}}\left(\omega H\right)$$

### Hygroscopicity and Liquid Retention Measurement

Liquid retention of different PAHL_3‐9_ samples (Φ = 15 mm, m≈1.0 g) was quantified by storing and weighing them at regular intervals for 100 days at ambient conditions (*T*_air_ = 18±2 °C, RH = 40±10%; *T*_dry_ = 18±2 °C, RH = 14%).

Liquid retention in different PAHL_4_W_x_ samples (Φ = 15 mm, m≈1.0 g) was quantified by storing and weighing them at regular intervals for 100 days at ambient conditions (*T*_air_ = 15±5 °C, RH = 45±10%; *T*_dry_ = 15±5 °C, RH = 14%).

### Thermal Properties and Thermal Cycling Stability Test

The thermal conductivity of PAHL samples was tested using a flash thermal conductivity tester (LFA467, Netzsch, Germany) at 25 °C and 45 °C corresponding to the crystalline and molten state, respectively. The melting temperature (*T*_m_), crystallization temperatures (*T*_c_) and phase change enthalpies (Δ*H*_m_, Δ*H*_c_) of the LNH, PAHL, and PAHLW samples were measured using differential scanning calorimetry (DSC, Q20, TA, USA) performed in N_2_ atmosphere with heating/cooling rate of 10 °C min^−1^ from −40 °C to 70 °C, and then cooling from 70 °C to −40 °C, with second heating to eliminate the thermal history. The DSC data reported in this study were obtained through necessary calculation by TA Universal Analysis software.

The LNH, PAHL, and PAHLW samples were heated to 70 °C (> *T*_m_) and then cooled to −40 °C (< *T*_c_) by adjusting the heating controller for 100 and 300 thermal cycles continuously. After the above thermal cycles, the thermal performance parameters were measured by DSC to evaluate the thermal cycling stability.

### Rheological and Compression Measurements

Rheological characterization of the prepared formulations was conducted using a modular intelligent rotary rheometer (MCR302, Anton Paar). The oscillation shear measurements of the PAHL samples (Φ = 25 mm, h = 2−3 mm) were carried out at 45 °C with an amplitude of 0.1% and an angular frequency from 100 to 0.1 rad s^−1^.

Compressive testing was performed with the INSTRON universal material experimental machine (Model 5967, Instron, USA) to obtain stress–strain curves and elastic modulus of crystalline and molten state PAHL samples (Φ = 15 mm, h = 20 mm). The pressure was determined by an instrument at a speed of 2 mm min^−1^ at room temperature.

### Adhesion Experiment

The interfacial shear adhesion strength was measured by a lap‐shear test. The substrates (PVC and PET) were washed with DI water and EtOH for 3 times and then dried. The PAHL salogels with a size of 30 mm (length) × 20 mm (width) × 1 mm (thickness) were clamped by two substrates. The devices were pressed with a pressure of ≈5 kPa and then cooled to allow PAHL_3_ crystallization. Finally, the devices were applied for the lap‐shear adhesion test. For the lap‐shear adhesion test, the tensile speed was 5 mm·min^−1^. The reported statistical data were obtained from at least five repeats of the same substrates. The interfacial shear adhesion strength (*F*_a_) was determined by dividing the peak force (*F*_peak_) by the overlap area (A):

(2) $$F_{a}=\frac{F_{peak}}{A}$$

To explore the adhesion and take‐off capability from skin, a rectangular PAHL_3_ sample (115 mm × 75 mm × 2 mm) was attached to the arm, performed stretching, bending, and tearing actions, and record the adhesion between the PAHL_3_ sample and skin using a digital camera.

### Thermal Management Test

The temperature variation of air, LNH, and PAHL salogels samples were measured by a thermocouple thermometer (HT‐9815, Dongguan Xintai Instrument Co., Ltd., China; resolution of 0.1 °C and accuracy of ±1 °C) under the condition of water bath heating and ice water bath cooling. 10 mL bottles containing air, or 5 g of the LNH, or 5 g of PAHL (PAHL_3_, PAHL_4_, PAHL_5,_ respectively) were put in a water bath, and each bottle was collected with a thermocouple thermometer. And the temperature‐time curves are recorded in Figure 5c,d. To compare the cooling effect, PAHL_3_ paste sample and two commercial antipyretic patch samples (with the same size 120 mm × 50 mm × 2 mm in length × width × thickness) were applied around the outer wall of a beaker containing 100 mL of 40 °C water at ambient temperature (*T*_air_ = 25 °C, RH = 30%), and compared their cooling rates. And the temperature–time curves are recorded in Figure 5e.

An infrared thermal imager (TiS50, Fluke, USA) was used to record the temperature variety of PAHL samples (Φ = 25 mm, h = 2−3 mm) on the 60 °C thermostatic platform and then on the −10 °C cooling platform, respectively.

PMMA model sun houses were designed from 3D printing and attached the 2 mm thick PAHL to the surface of house and recorded the temperature variation inside the house using a thermocouple thermometer under simulated sunlight exposure (110 mW cm^−2^, *T*_air_ = 25 °C, RH = 45%, illustrated in Figure S15, Supporting Information), using the model house without PAHL patches as a blank control. The cooling process was carried out in a cooling chamber (*T*_air_ = −18 °C, RH = 30%).

### Optical Transparency Test

UV–vis transmittance measurement was performed on a UV 3600 (Shimadzu Corporation, Japan) with a wavelength from 200 to 800 nm. Before the test, pour the salogel prepolymer into the quartz cuvette (722/10 mL), irradiate by 365 nm UV light for 20 s for polymerization.

### Frost‐Resistance and Ice‐Blocking Measurements

A cooled thermoelectric cold plate capable of lowering the temperature from 20 °C to −18 °C within 5 min was used for all the condensation‐frosting experiments. The Peltier surface temperature (*T*_pel_) was controlled using a temperature controller. The experiments were recorded using a Sony camera.

In a copper sheet (30 × 50 mm), 3 g PAHL gel film was rapidly cooled to few degrees below their *T*_m_ in controlled environmental conditions (*T*_pel_ = −18 °C; RH = 38%; *T*_air_ = 25 °C) forming a 3 mm thick solidified film. The bared copper surface without coating was used as the control group. The ice block was quickly placed on the upper surface and tilted it 15°, and recorded the optical images with the camera.

### Density Functional Theory Calculations

The interaction strength and free energy calculations were performed with the Gaussian 09 program and the B3LYP/6‐31G^**^ method was used to optimize geometric structures. The model of LNH was composed of three components (i.e., Li^+^, NO_3_^−^ and H_2_O) and the model of P(AM‐*co*‐HEMA) hydrogel was composed of three monomers (i.e., AM, HEMA, and H_2_O). The interaction energy, *E*_int_, between the components in the system is calculated as:

(3) $$E_{\mathrm{int}}=E_{\mathrm{total}}-\sum E_{\mathrm{component}}$$

where *E*_total_ and *E*_component_ are the total energy of the whole system and the energy of each component in the system, respectively. Based on the above definition, a negative *E*_int_ corresponds to stable interaction between the components, and the more negative *E*_int_ indicates a stronger interaction in the system. To eliminate the dispersion effect, the keyword of empirical dispersion = gd3bj in the route section was adopted in the simulation process, and all calculations of interaction strength were optimized without symmetric restrictions.

Specially, in the solute phase, the implicit solvent approaches IEF‐PCM (polarizable continuum model) method was used as continuum solvation models.^[^ ^50^, ^51^ ^]^ The IEF‐PCM model is based on considering the solvent as a continuous medium and calculating the solvation energy components of a solute placed in the cavity of this medium by the SCRF procedure. Prior to SCRF computations, all solute geometries were optimized in vacuo at the B3LYP/6‐31G^**^ level of theory. The model of P(AM‐*co*‐HEMA) polymer chains was composed of three monomer units including two AM and one HEMA unit according to the molar ratio of the PAH hydrogels. The solvation‐free energy (Δ*G*_solv_) is the difference in free energy of the solute in the solution (*G*_soln_) and gas phases (*G*_gas_):^[^ ^51^ ^]^

(4) $$\Delta \;G_{\mathrm{solv}}=G_{\mathrm{soln}}-G_{\mathrm{gas}}$$

## Conflict of Interest

The authors declare no conflict of interest.

## Author Contributions

C.Y. carried out the synthetic work, conducted material characterizations, and finished the simulation work and original draft preparation. J.S. and C.C. performed the thermal management experiments and Rheological and Compression Measurements. K.Y. and Y.L. performed review & editing. L.Y.S. designed and supervised the study and wrote the manuscript.

## Supporting information

Supporting Information

## Data Availability

The data that support the findings of this study are available from the corresponding author upon reasonable request.

## References

[1] J. Woods , A. Mahvi , A. Goyal , E. Kozubal , A. Odukomaiya , R. Jackson , Nat. Energy 2021, 6, 295.

[2] S. A. Mohamed , F. A. Al‐Sulaiman , N. I. Ibrahim , M. H. Zahir , A. Al‐Ahmed , R. Saidur , B. S. Yılbaş , A. Z. Sahin , Renewable Sustinable Energy Rev. 2017, 70, 1072.

[3] A. Usman , F. Xiong , W. Aftab , M. Qin , R. Zou , Adv. Mater. 2022, 34, 2202457. 10.1002/adma.202202457 35616900

[4] G.‐Z. Yin , J. Hobson , Y. Duan , D.‐Y. Wang , Energy Storage Mater. 2021, 40, 347.

[5] S. Kiyabu , P. Girard , D. J. Siegel , J. Am. Chem. Soc. 2022, 144, 21617. 36394989 10.1021/jacs.2c08993

[6] P. A. J. Donkers , L. C. Sögütoglu , H. P. Huinink , H. R. Fischer , O. C. G. Adan , Appl. Energy 2017, 199, 45.

[7] M. H. Mruthunjayappa , N. S. Kotrappanavar , D. Mondal , Prog. Mater. Sci. 2022, 126, 100932.

[8] B. B. Hansen , S. Spittle , B. Chen , D. Poe , Y. Zhang , J. M. Klein , A. Horton , L. Adhikari , T. Zelovich , B. W. Doherty , B. Gurkan , E. J. Maginn , A. Ragauskas , M. Dadmun , T. A. Zawodzinski , G. A. Baker , M. E. Tuckerman , R. F. Savinell , J. R. Sangoro , Chem. Rev. 2021, 121, 1232. 33315380 10.1021/acs.chemrev.0c00385

[9] C. Liu , L. Wei , X. Yin , X. Pan , J. Hu , N. Li , J. Xu , J. Jiang , K. Wang , Chem. Eng. J. 2021, 425, 130608.

[10] R. D. Rogers , K. R. Seddon , Science 2003, 302, 792. 14593156 10.1126/science.1090313

[11] T. P. Lodge , T. Ueki , Acc. Chem. Res. 2016, 49, 2107. 10.1021/acs.accounts.6b00308 27704769

[12] P. Karimineghlani , E. Emmons , M. J. Green , P. Shamberger , S. A. Sukhishvili , J. Mater. Chem. A 2017, 5, 12474.

[13] P. Karimineghlani , A. Palanisamy , S. A. Sukhishvili , ACS Appl. Mater. Interfaces 2018, 10, 14786. 29633618 10.1021/acsami.8b03080

[14] K. K. Rajagopalan , P. Karimineghlani , X. Zhu , P. J. Shamberger , S. A. Sukhishvili , J. Mater. Chem. A 2021, 9, 25892.

[15] C. Yin , J. Lan , X. Wang , Y. Zhang , R. Ran , L.‐Y. Shi , ACS Appl. Mater. Interfaces 2021, 13, 21810. 33905220 10.1021/acsami.1c03996

[16] Y. Zhang , S. Furyk , D. E. Bergbreiter , P. S. Cremer , J. Am. Chem. Soc. 2005, 127, 14505. 16218647 10.1021/ja0546424

[17] Y. Zhang , P. S. Cremer , Curr. Opin. Chem. Biol. 2006, 10, 658. 17035073 10.1016/j.cbpa.2006.09.020

[18] Y. Marcus , Chem. Rev. 2009, 109, 1346. 19236019 10.1021/cr8003828

[19] S. Xie , A. Nikolaev , O. A. Nordness , L. C. Llanes , S. D. Jones , P. M. Richardson , H. Wang , R. J. Clément , J. Read de Alaniz , R. A. Segalman , Macromolecules 2022, 55, 5723.

[20] K. W. Gao , X. Yu , R. M. Darling , J. Newman , N. P. Balsara , Soft Matter 2022, 18, 282. 34918729 10.1039/d1sm01511g

[21] Y. Li , T. Wei , L. Chen , K. Wang , Y. Shi , J. Hazard Mater. 2022, 427, 128203. 34999402 10.1016/j.jhazmat.2021.128203

[22] N. Molinari , J. P. Mailoa , B. Kozinsky , Chem. Mater. 2018, 30, 6298.

[23] J. Li , X. Wang , T. Lan , Y. Lu , M. Hong , L. Ding , L. Wang , Exp. Biol. Med. 2022, 11, 858. 10.3390/biology11060858 PMC9220195 35741379

[24] J. C. Lai , Y. C. Kam , H. C. Lin , C. S. Wu , Comp. Biochem. Physiol., Part A: Mol. Integr. Physiol. 2019, 227, 84. 10.1016/j.cbpa.2018.09.025 30308302

[25] R. J. Tiika , J. Wei , G. Cui , Y. Ma , H. Yang , H. Duan , BMC Plant Biol. 2021, 21, 491. 34696719 10.1186/s12870-021-03269-y PMC8547092

[26] E. van Zelm , Y. Zhang , C. Testerink , Annu. Rev. Plant Biol. 2020, 71, 403. 32167791 10.1146/annurev-arplant-050718-100005

[27] W. Voigt , D. Zeng , Pure Appl. Chem. 2002, 74, 1909.

[28] S. B. Rempe , L. R. Pratt , G. Hummer , J. D. Kress , R. L. Martin , A. Redondo , J. Am. Chem. Soc. 2000, 122, 966.

[29] M. R. McCoustra , Phys. Chem. Chem. Phys. 2008, 10, 4676. 18688508 10.1039/b812223g

[30] C. Q. Sun , in Solvation Dynamics,Vol. 121, Springer, Berlin, New York 2019, pp. 19.

[31] Y. Wang , W. Zhu , K. Lin , L. Yuan , X. Zhou , S. Liu , J. Raman Spectrosc. 2016, 47, 1231.

[32] Y. Huang , Z. Ma , X. Zhang , G. Zhou , Y. Zhou , C. Q. Sun , J. Phys. Chem. B 2013, 117, 13639. 24090472 10.1021/jp407836n

[33] D. J. Goebbert , E. Garand , T. Wende , R. Bergmann , G. Meijer , K. R. Asmis , D. M. Neumark , J. Phys. Chem. A 2009, 113, 7584. 19445493 10.1021/jp9017103

[34] S. Kannan , N. Kumar , M. A. Jog , R. M. Manglik , Ind. Eng. Chem. Res. 2022, 61, 16341.

[35] D. G. Atinafu , B. Y. Yun , S. Yang , H. Yuk , S. Wi , S. Kim , Energy Storage Mater. 2021, 42, 164.

[36] Y. E. Milián , A. Gutiérrez , M. Grágeda , S. Ushak , Renewable Sustainable Energy Rev. 2017, 73, 983.

[37] B. K. Purohit , V. S. Sistla , Energy Storage 2020, 3, 212.

[38] X. Huang , X. Chen , A. Li , D. Atinafu , H. Gao , W. Dong , G. Wang , Chem. Eng. J. 2019, 356, 641.

[39] W. Aftab , X. Huang , W. Wu , Z. Liang , A. Mahmood , R. Zou , Energy Environ. Sci. 2018, 11, 1392.

[40] W. Gao , J. Chang , X. Li , S. Li , Y. Zhou , X. Hou , L. Long , J. Zhao , X. Yuan , Adv. Funct. Mater. 2023, 33, 2303306.

[41] T. Zhu , C. Jiang , M. Wang , C. Zhu , N. Zhao , J. Xu , Adv. Funct. Mater. 2021, 31, 2102433.

[42] Y. Fang , X. Xiong , L. Yang , W. Yang , H. Wang , Q. Wu , Q. Liu , J. Cui , Adv. Funct. Mater. 2023, 33, 2301505.

[43] X. Liu , L. Zhang , B. El Fil , C. D. Díaz‐Marín , Y. Zhong , X. Li , S. Lin , E. N. Wang , Adv. Mater. 2023, 35, 2211763. 10.1002/adma.202211763 36921061

[44] L. Xu , Y. Qiao , D. Qiu , Adv. Mater. 2023, 35, 2209913. 10.1002/adma.202209913 36628947

[45] S. Huang , L. Hou , T. Li , Y. Jiao , P. Wu , Adv. Mater. 2022, 34, 2110140. 10.1002/adma.202110140 35122340

[46] Y. Cui , Y. Ke , C. Liu , Z. Chen , N. Wang , L. Zhang , Y. Zhou , S. Wang , Y. Gao , Y. Long , Joule 2018, 2, 1707.

[47] S. Liu , Y. Li , Y. Wang , K. M. Yu , B. Huang , C. Y. Tso , Adv. Sci. 2022, 9, 2106090. 10.1002/advs.202106090 PMC9108621 35486020

[48] C. Lin , J. Hur , C. Y. H. Chao , G. Liu , S. Yao , W. Li , B. Huang , Sci. Adv. 2022, 8, 7359. 10.1126/sciadv.abn7359 PMC9054005 35486733

[49] P. Khomein , A. Nallapaneni , J. Lau , D. Lilley , C. Zhu , S. Kaur , R. Prasher , G. Liu , Sol. Energy Mater. Sol. Cells 2021, 225, 111030.

[50] A. Alibakhshi , B. Hartke , Nat. Commun. 2021, 12, 3584. 34145237 10.1038/s41467-021-23724-6 PMC8213834

[51] J. Ho , A. Klamt , M. L. Coote , J. Phys. Chem. A 2010, 114, 13442. 21133342 10.1021/jp107136j

