# Supplementary material for: Chaotropic Ions Mediated Polymer Gelation for Thermal Management
Source: Adv Sci (Weinh). 2024 Jul 3;11(32):2405077. doi: 10.1002/advs.202405077 (PMC11348148; doi:10.1002/advs.202405077)
Supplement: Supplementary file 1 — Supporting Information [file ADVS-11-2405077-s001.docx]

**Supporting Information**

**Chaotropic Ions Mediated Polymer Gelation for Thermal Management**

*Chenxiao Yin,^†^* *Jingrui Sun,^†^ Chang Cui,^†^ Ke-Ke Yang,^‡^ Ling-Ying Shi,^†,*^ Yiwen Li^†^*

^†^ College of Polymer Science and Engineering, Sichuan University, and State Key Laboratory of Polymer Materials Engineering, Chengdu 610065, China.

^‡^ The Collaborative Innovation Center for Eco-Friendly and Fire-Safety Polymeric Materials (MoE), National Engineering Laboratory of Eco-Friendly Polymeric Materials (Sichuan), State Key Laboratory of Polymer Materials Engineering, College of Chemistry, Sichuan University, Chengdu 610064, China.

*** To whom correspondence should be addressed. Email: shilingying@scu.edu.cn

**1. Synthesis of** **Lithium Nitrate Trihydrate (LiNO_3_⋅H_2_O, LNH) and Lithium Nitrate Trihydrate aqueous solution (LNHW) as well as other salt hydrates**

Molten LNH were prepared by dissolving anhydrous lithium nitrate and deionized water (m (LiNO_3_): m (H_2_O) = 56.06 : 43.94) according to the stoichiometric ratio at room conditions. Post addition, the solution container was sealed, and the mixture was stirred for 20 min to ensure complete dissolution resulting in a transparent solution.

Henceforth, LNHW_x_ were prepared by adding varying quantities of H_2_O (2.5, 5, 10, and 20 wt% to LNH) in a pre-measured amount of LNH at room conditions. For example, LNHW_4_ denotes to add 20 wt% H_2_O into the LNH solution.

Molten CCH were prepared by dissolving anhydrous calcium chloride and deionized water (m(CaCl_2_): m(H_2_O) = 50.66 : 49.34) according to the stoichiometric ratio at room conditions.

**2. FTIR** **analysis of the Lithium Nitrate Trihydrate (LiNO_3_⋅H_2_O, LNH)**

The stretching vibration of −OH is sensitive to changes in hydrogen bonding types. There is a significant difference in the −OH characteristic peaks of water and LNH in FTIR (Fig. S4a), in which the −OH stretching vibration can be deconvoluted into strong HBs, weak HBs, and free −OH, including the four HBs vibration modes^[1,2]^ shown in Fig. S4d. Compared with pure water ^[2]^, the strong HBs band intensity of LNH significantly decreases, while the bifurcated HBs band of NO_3_^−^ and H_2_O significantly increases^[3]^ (Fig. S4b-c).

**Table S1.** The molar concentration of SSD (Na_2_SO_4_⋅10H_2_O), DPDH (Na_2_HPO_4_⋅12H_2_O), CCH (CaCl_2_⋅6H_2_O), and LNH (LiNO_3_⋅3H_2_O).

| Salt hydrates | SSD | DPDH | CCH | LNH |
| --- | --- | --- | --- | --- |
| Concentration (M) | 5.6 | 4.6 | 9.2 | 18.5 |

**Table S2**. The detailed raw materials of P(Am-*co*-HEMA))/LNH (PAHL_y_, y = 1−10).

| Samples | AM (mmol) | HEMA (mmol) | LNH (g) | 1173 (mmol) |
| --- | --- | --- | --- | --- |
| PAHL_1_ | 0.703 | 0.768 | 2 | 0.012 |
| PAHL_2_ | 1.407 | 0.384 | 2 | 0.015 |
| PAHL_3_ | 1.407 | 0.768 | 2 | 0.018 |
| PAHL_4_ | 1.407 | 1.537 | 2 | 0.024 |
| PAHL_5_ | 2.814 | 0.768 | 2 | 0.029 |
| PAHL_6_ | 1.407 | 2.305 | 2 | 0.030 |
| PAHL_7_ | 2.814 | 2.305 | 2 | 0.042 |
| PAHL_8_ | 1.407 | 3.074 | 2 | 0.037 |
| PAHL_9_ | 2.814 | 3.074 | 2 | 0.048 |
| PAHL_10_ | 1.407 | 3.842 | 2 | 0.043 |

**Table S3.** The detailed raw materials of P(Am-*co*-HEMA))/LNHW (PAHL_4_W_x_, x=1−4).

| Samples | AM  (mmol) | HEMA  (mmol) | LNH  (g) | H_2_O  (g) | 1173  (mmol) |
| --- | --- | --- | --- | --- | --- |
| PAHL_4_W_1_ | 1.407 | 1.537 | 2 | 0.05 | 0.024 |
| PAHL_4_W_2_ | 1.407 | 1.537 | 2 | 0.1 | 0.024 |
| PAHL_4_W_3_ | 1.407 | 1.537 | 2 | 0.2 | 0.024 |
| PAHL_4_W_4_ | 1.407 | 1.537 | 2 | 0.4 | 0.024 |


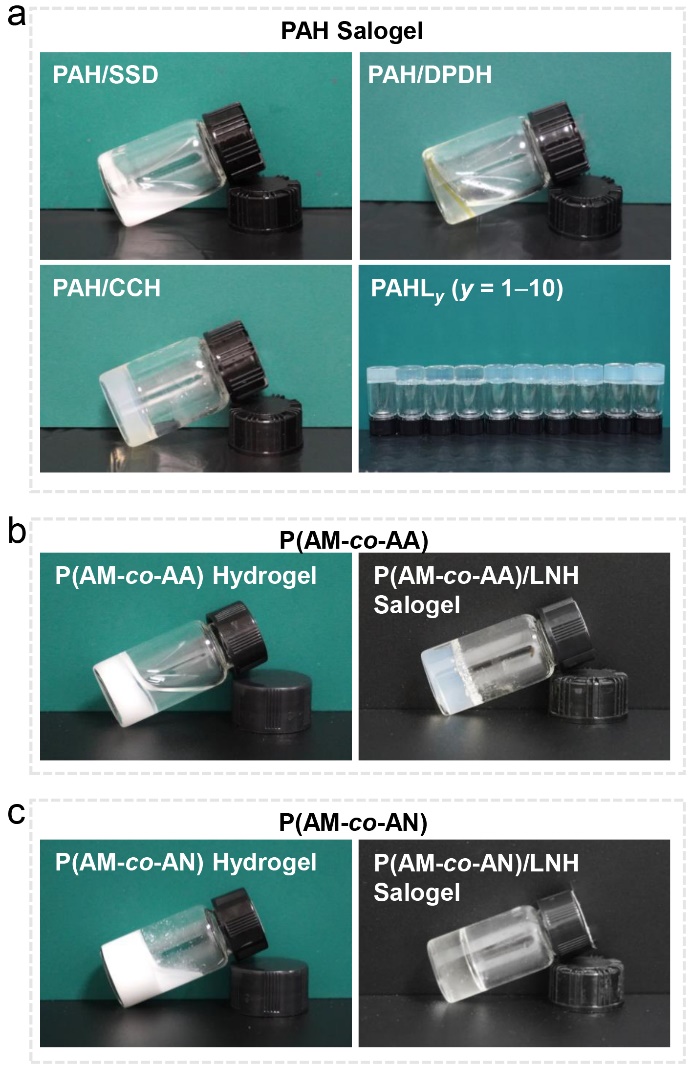


**Fig. S1** The photographs of (a) PAH salogels, including PAH/SSD, PAH/DPDH, PAH/CCH, and PAHL_1−10_; (b) P(AM-*co*-AA) hydrogels and P(AM-*co*-AA)/LNH salogels; (c) P(AM-*co*-AN) hydrogels and P(AM-co-AN)/LNH salogels.


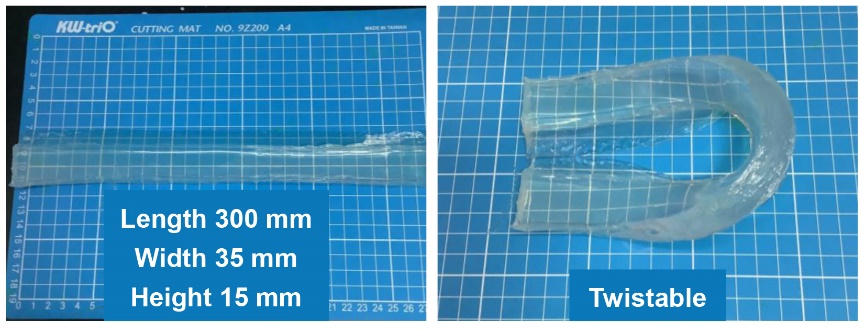


**Fig. S2** The photographs of bulky PAHL_3_ samples.


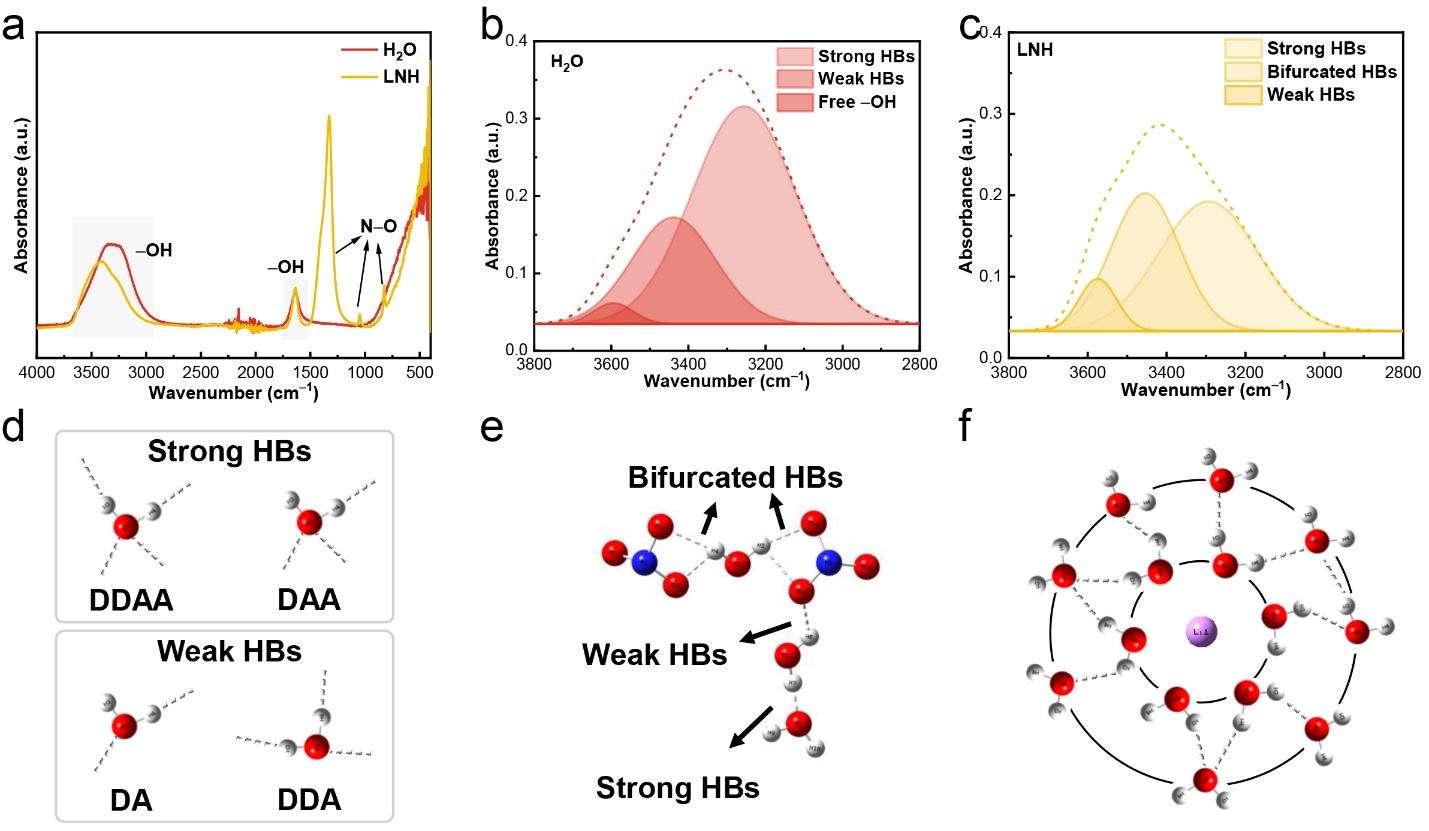


**Fig. S3** (a)The FT-IR spectra of water and LNH. The −OH stretching vibration of (b) water and (c) LNH were deconvoluted into three sub-bands. (d) Hydrogen bond vibration mode of water. The solvation of (e) NO_3_^−^ and (f) Li^+^ in water.


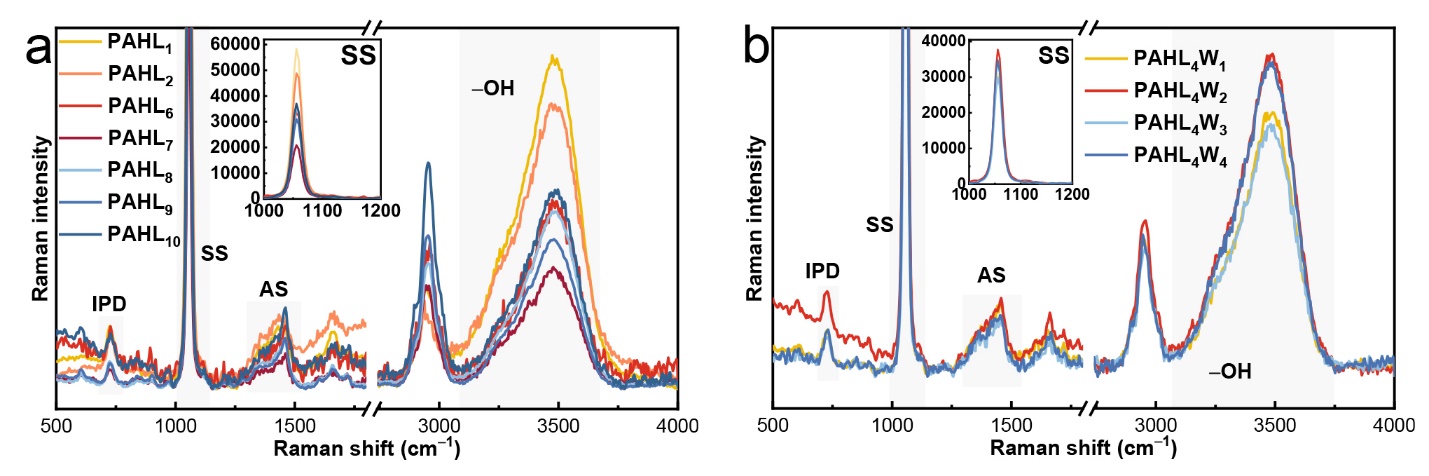


**Fig. S4** The Raman spectra of (a) PAHL_1−2_ and PAHL_6−10_, and (b) PAHL_4_W_1−4_.


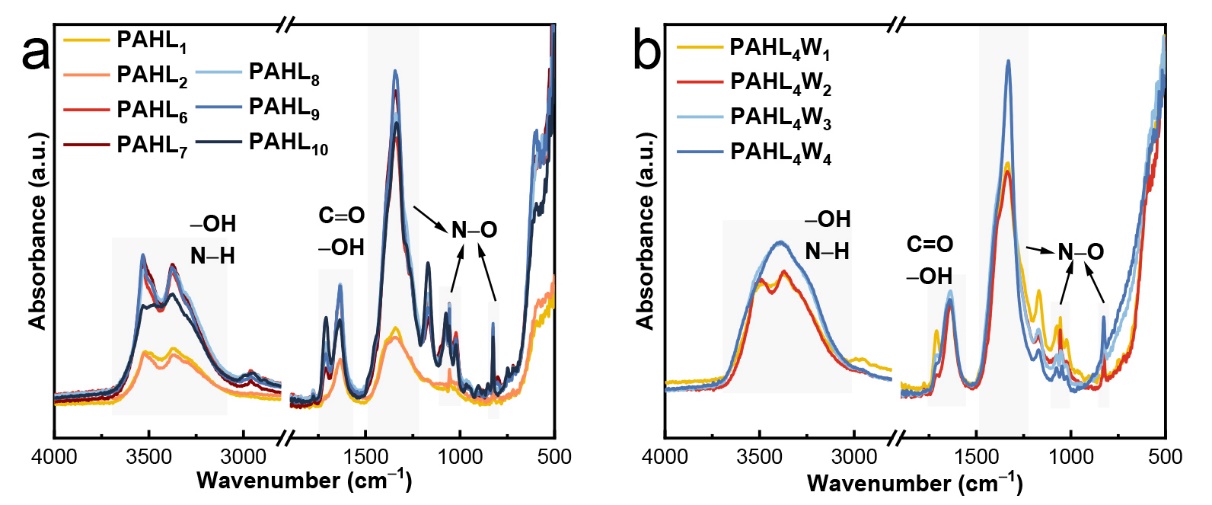


**Fig. S5** The FT-IR spectra of (a) PAHL_1−2_ and PAHL_6−10_, and (b) PAHL_4_W_1−4_.

**Table S4.** The variation of the *f*_whm_ (peak width) of SS band in Raman spectra for LNH and PAHL_1−3_.

| Samples | LNH | PAHL_1_ | PAHL_2_ | PAHL_3_ |
| --- | --- | --- | --- | --- |
| *f*_whm_ (cm^−1^) | 371 | 220 | 228 | 216 |


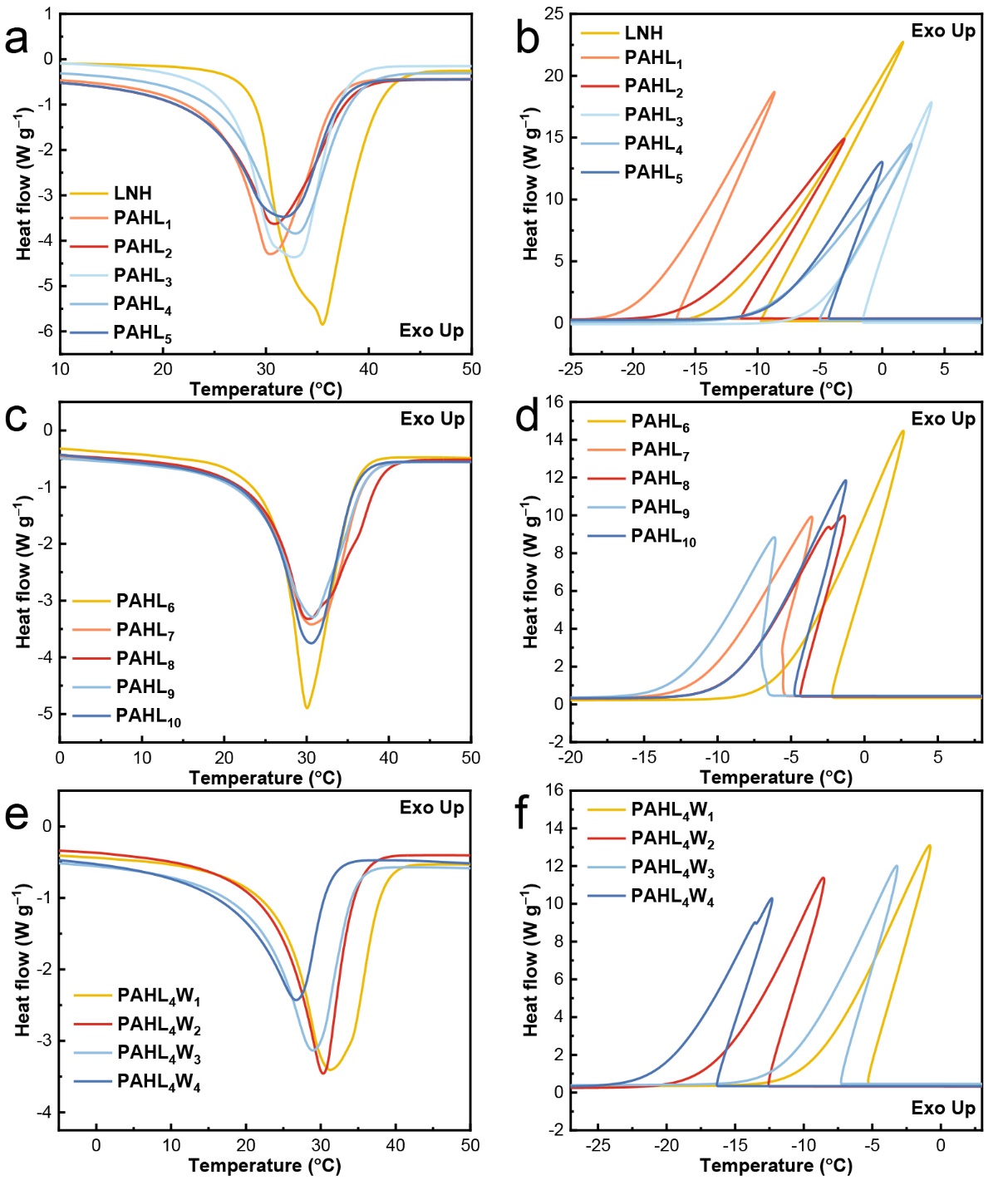


**Fig. S6** The DSC curves of PAHL_1−10_ and PAHL_4_W_1−4_.

**Table S5.** The detailed phase change parameters i.e. phase change temperature (*T*_m_, *T*_c_), phase transition enthalpy (Δ*H*_m_, Δ*H*_c_) and of LNH and PAHL.

| Samples | | *T*_m_ (^o^C) | | *T*_m_^p^ (^o^C) | | Δ*H*_m_ (J g^−1^) | | *T*_c_ (^o^C) | *T*_c_^p^ (^o^C) | Δ*H*_c_ (J g^−1^) | |
| --- | --- | --- | --- | --- | --- | --- | --- | --- | --- | --- | --- |
| LNH | | 28.9 | | 35.5 | | 263.1 | | −8.8 | 1.7 | 233.0 | |
| PAHL_1_ | | 25.5 | | 30.4 | | 205.4 | | −16.1 | −8.7 | 185.7 | |
| PAHL_2_ | 24.5 | | 30.9 | | 208.1 | | −3.1 | | −10.6 | | 194.0 |
| PAHL_3_ | 26.4 | | 32.7 | | 216.5 | | −1.2 | | 3.9 | | 200.5 |
| PAHL_4_ | 25.2 | | 32.9 | | 216.2 | | −4.2 | | 2.3 | | 196.2 |
| PAHL_5_ | 23.7 | | 31.8 | | 211.1 | | −3.8 | | 0 | | 194.0 |
| PAHL_6_ | | 26.1 | | 30.1 | | 187.0 | | −1.5 | 2.6 | 172.3 | |
| PAHL_7_ | | 24.2 | | 30.5 | | 183.0 | | −5.8 | −3.6 | 162.3 | |
| PAHL_8_ | | 24.5 | | 30.2 | | 192.4 | | −4.2 | −1.4 | 175.1 | |
| PAHL_9_ | | 23.4 | | 30.8 | | 173.9 | | −7.3 | −6.1 | 159.5 | |
| PAHL_10_ | | 24.4 | | 30.6 | | 177.1 | | −4.8 | −1.72 | 163.0 | |

**Table S6.** The detailed phase change parameters i.e. phase change temperature (*T*_m_, *T*_c_), phase transition enthalpy (Δ*H*_m_, Δ*H*_c_) and of PAHLW.

| Samples | *T*_m_ (^o^C) | *T*_m_^p^ (^o^C) | Δ*H*_m_ (J g^−1^) | *T*_c_ (^o^C) | *T*_c_^p^ (^o^C) | Δ*H*_c_ (J g^−1^) |
| --- | --- | --- | --- | --- | --- | --- |
| PAHL_4_W_1_ | 24.3 | 31.3 | 196.4 | −4.8 | −0.8 | 182.7 |
| PAHL_4_W_2_ | 23.6 | 30.3 | 170.5 | −11.8 | −8.6 | 160.1 |
| PAHL_4_W_3_ | 21.0 | 28.9 | 170.9 | −6.6 | −3.2 | 164.8 |
| PAHL_4_W_4_ | 17.2 | 26.7 | 156.6 | −15.9 | −12.3 | 142.5 |


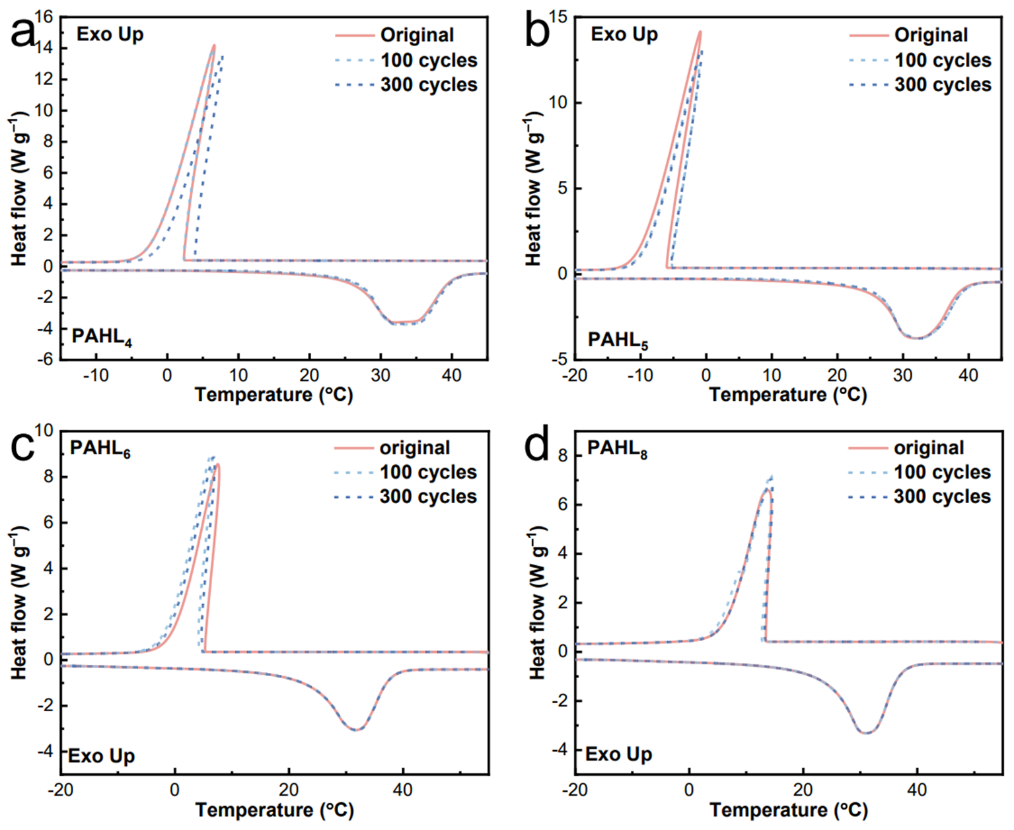


**Fig. S7** The DSC curves of PAHL_4−6_, and PAHL_8_ after 100 and 300 thermal cycles.


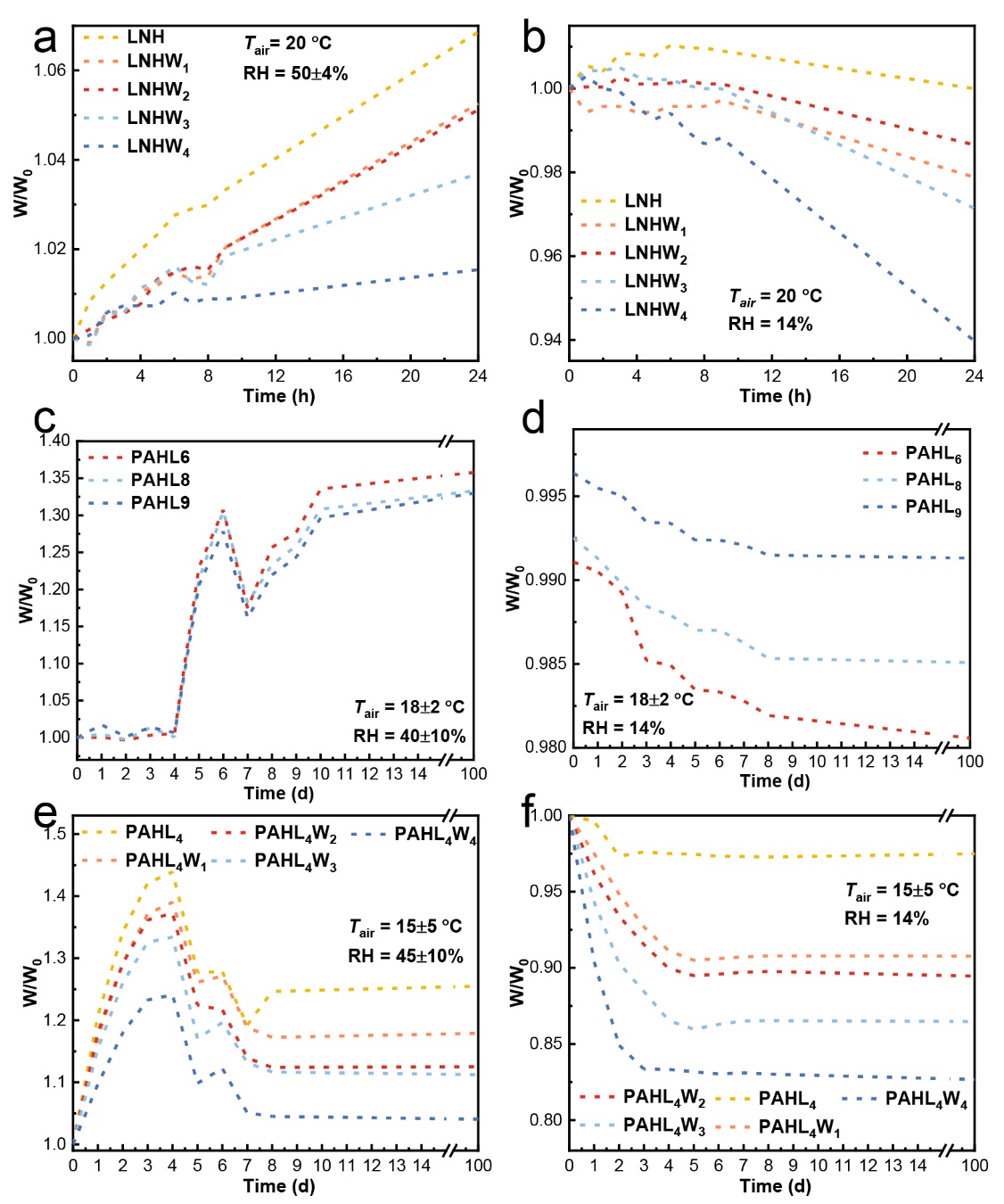


**Fig. S8** The liquid retention of PAHL_3−5_ samples at (a) ambient conditions (*T*_air_ = 18±2 °C, RH = 40±10%) and (b) dry conditions (*T*_dry_ = 18±2 °C, RH = 14%). The water absorption/loss rate of PAHL_6, 8, and 9_ at (c) ambient conditions (*T*_air_ = 18±2 °C, RH = 40±10%) and (d) dry conditions (*T*_dry_ = 18±2 °C, RH = 14%). The water absorption/loss rate of PAHL_4_W_1−4_ at (e) ambient conditions (*T*_air_ = 15±5 °C, RH = 45±10%) and (f) dry conditions (*T*_dry_ = 15±5 °C, RH = 14%).


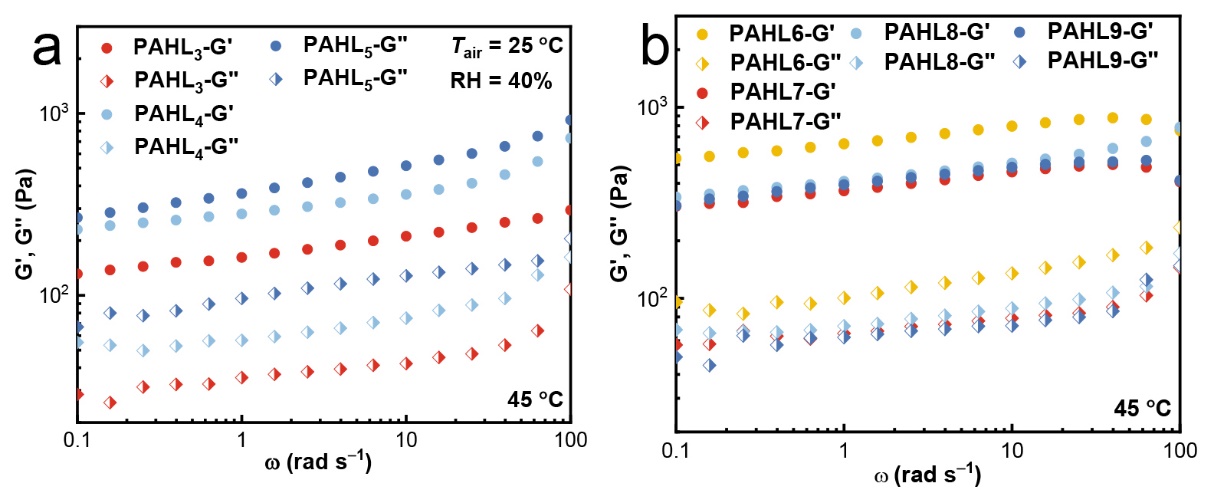


**Fig. S9** The oscillation shear measurements of the (a) PAHL_3−5_ and (b) PAHL_6−9_ at 45 °C.


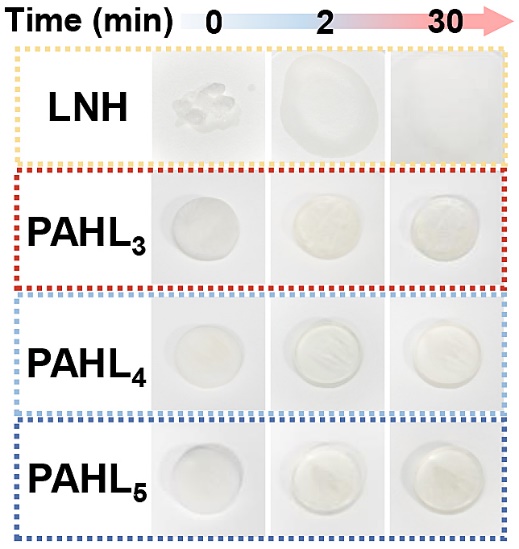


**Fig. S10** Form-stable measurement. The photos of LNH and the PAHL_3−5_ samples heating on a 60 ^o^C thermostatic platform.


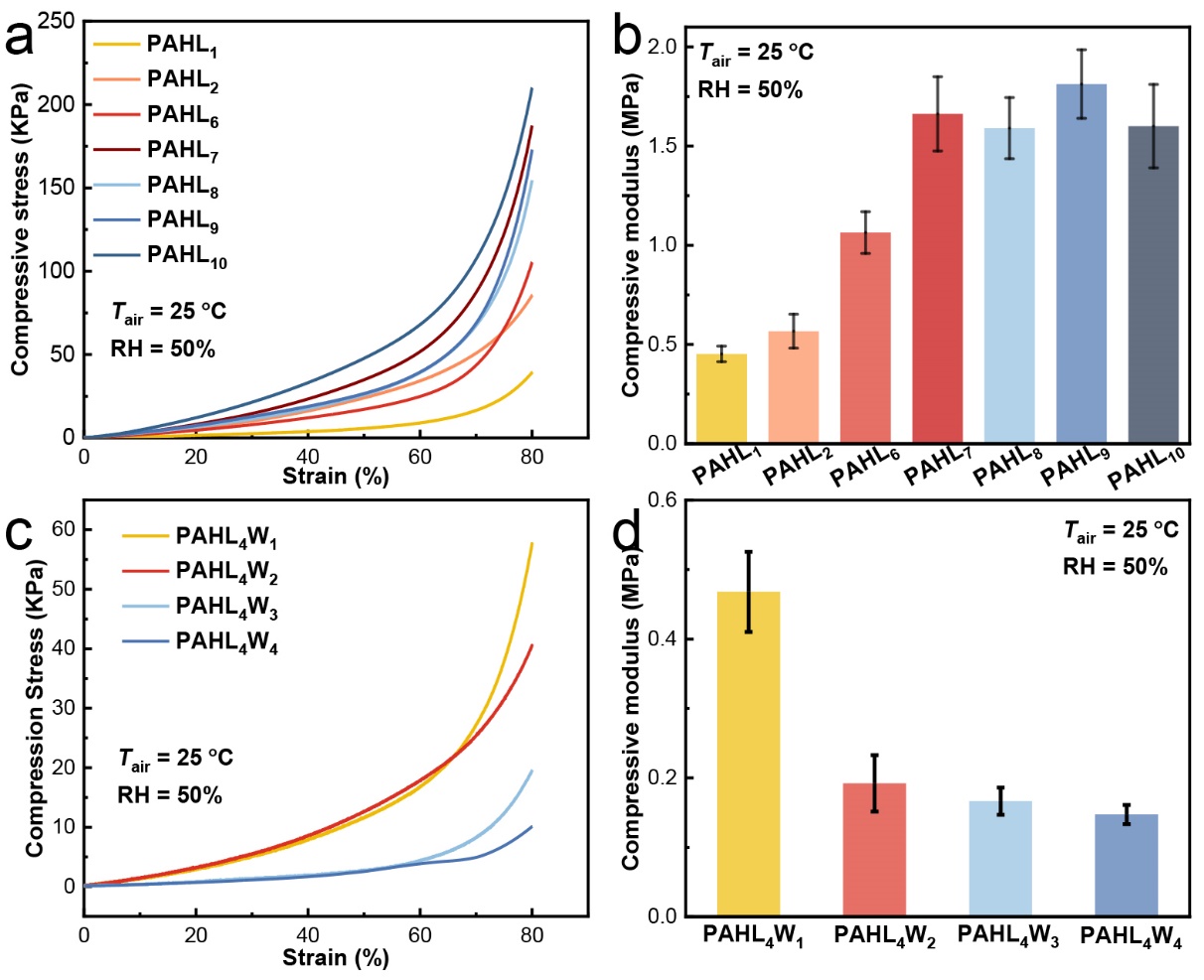


**Fig. S11** The compression stress-strain curves and elastic modulus of PAHL_1,2, and 6−10_ and PAHL_4_W_1−4_ in molten state.


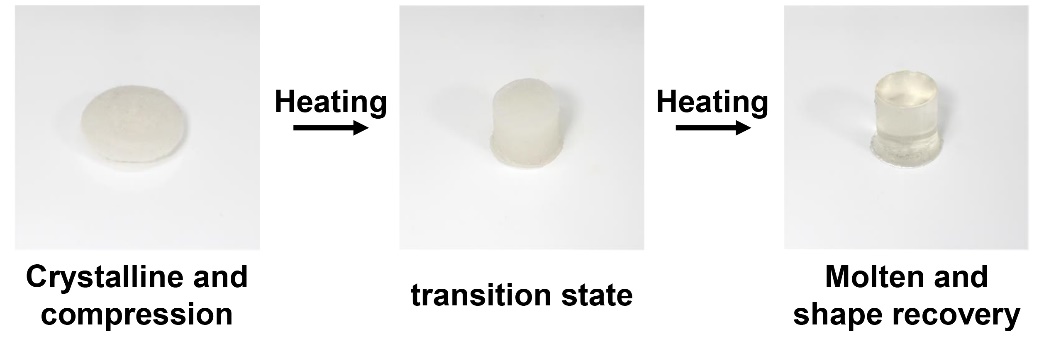


**Fig. S12** photos show that PAHL can recover its initial elastic state by heating after crystallization and compression.


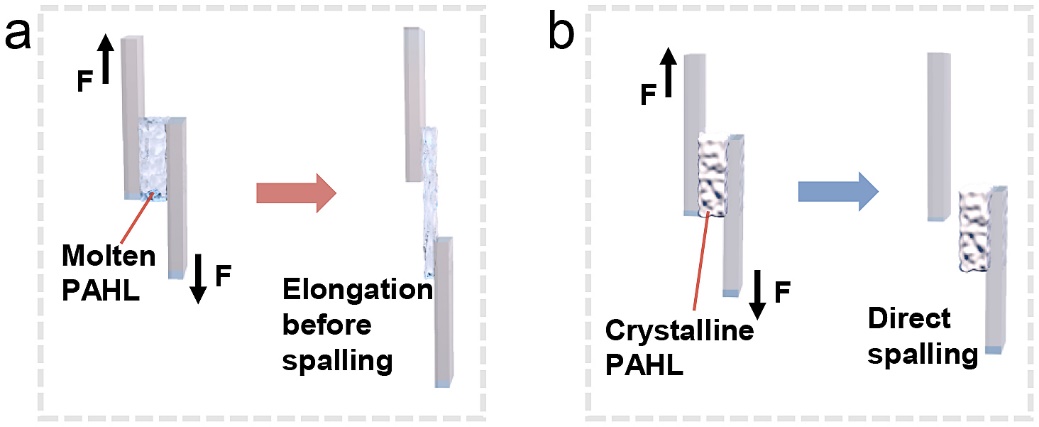


**Fig. S13** Schematic diagram of lap-shear adhesion test for molten and crystalline PAHL.


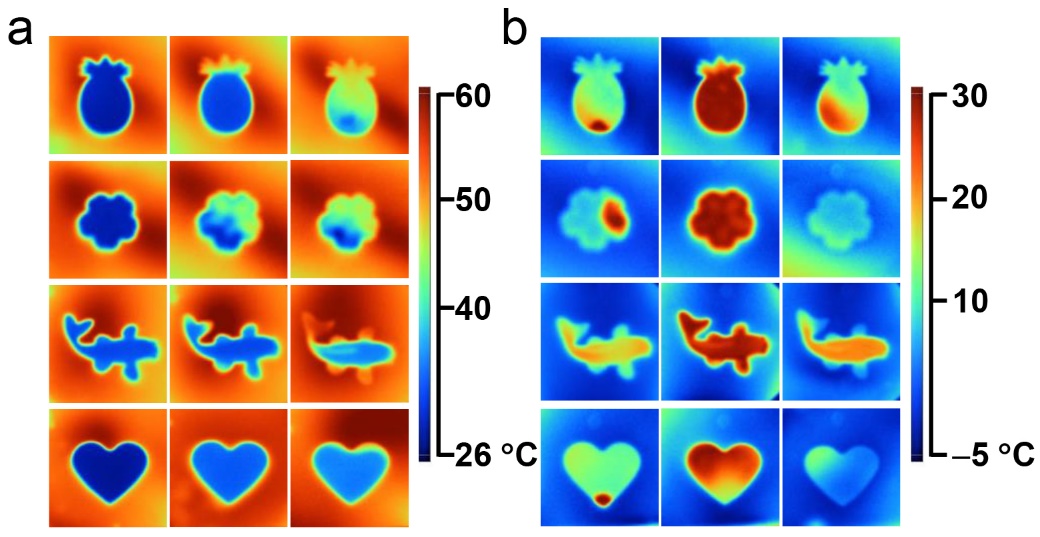


**Fig. S14** The infrared thermal images of temperature varieties for PAHL_3_ samples with different shapes on the (a) 60 ^o^C thermostatic platform and (b) −10 ^o^C cooling platform.


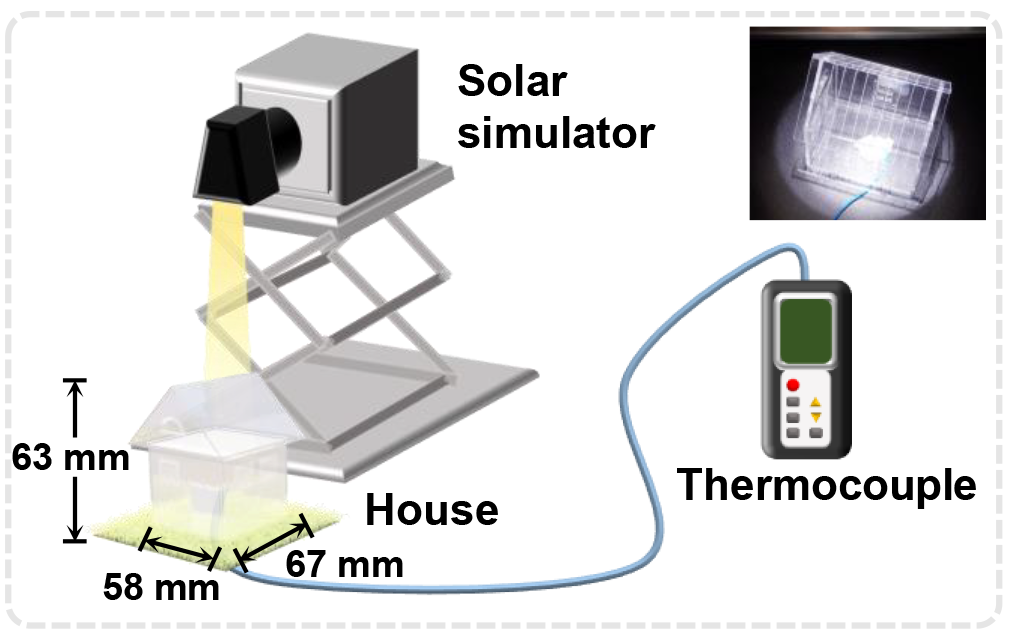


**Fig. S15** The experimental testing device for sunroom. Place the sun room under the solar simulator (110 mw cm^−2^) and connect the thermocouple to record the temperature change.

**References**

[1] Sun, Q. The Raman OH stretching bands of liquid water. *Vibrational Spectroscopy* **51**, 213-217, doi:10.1a016/j.vibspec.2009.05.002 (2009).

[2[ Ludwig, R. Water: From Clusters to the Bulk. *Angewandte Chemie International Edition* **40**, 1808-1827, doi:10.1002/1521-3773(20010518)40:10<1808::Aid-anie1808>3.0.Co;2-1 (2001).

[3] Thogersen, J. *et al.* Hydration dynamics of aqueous nitrate. *J Phys Chem B* **117**, 3376-3388, doi:10.1021/jp310090u (2013).
